# The Influence of Hepatic Steatosis and Fibrosis on Postoperative Outcomes After Major Liver Resection of Perihilar Cholangiocarcinoma

**DOI:** 10.1245/s10434-023-14419-x

**Published:** 2023-10-29

**Authors:** Anne-Marleen van Keulen, Pim B. Olthof, Stefan Buettner, Jan Bednarsch, Joanne Verheij, Joris I. Erdmann, Lynn E. Nooijen, Robert J. Porte, Robert C. Minnee, Sarwa Darwish Murad, Ulf P. Neumann, Lara Heij, Bas Groot Koerkamp, Michail Doukas

**Affiliations:** 1 Department of Surgery, Erasmus MC Cancer Institute, Rotterdam, The Netherlands; 2 Department of Surgery and Transplantation, University Hospital RWTH Aachen, Aachen, Germany; 3 Department of Pathology, Cancer Center Amsterdam, Amsterdam University Medical Centers, Amsterdam, The Netherlands; 4 Department of Surgery, Amsterdam University Medical Centers, Amsterdam, The Netherlands; 5 Department of Gastroenterology and Hepatology, Erasmus MC University Medical Center, Rotterdam, The Netherlands; 6 Department of Surgery, Maastricht University Medical Center (MUMC), Maastricht, The Netherlands; 7 Department of Pathology, Erasmus MC, Rotterdam, The Netherlands

## Abstract

**Background:**

Surgical resection for perihilar cholangiocarcinoma (pCCA) is associated with high operative risks. Impaired liver regeneration in patients with pre-existing liver disease may contribute to posthepatectomy liver failure (PHLF) and postoperative mortality. This study aimed to determine the incidence of hepatic steatosis and fibrosis and their association with PHLF and 90-day postoperative mortality in pCCA patients.

**Methods:**

Patients who underwent a major liver resection for pCCA were included in the study between 2000 and 2021 from three tertiary referral hospitals. Histopathologic assessment of hepatic steatosis and fibrosis was performed. The primary outcomes were PHLF and 90-day mortality.

**Results:**

Of the 401 included patients, steatosis was absent in 334 patients (83.3%), mild in 58 patients (14.5%) and moderate to severe in 9 patients (2.2%). There was no fibrosis in 92 patients (23.1%), periportal fibrosis in 150 patients (37.6%), septal fibrosis in 123 patients (30.8%), and biliary cirrhosis in 34 patients (8.5%). Steatosis (≥ 5%) was not associated with PHLF (odds ratio [OR] 1.36; 95% confidence interval [CI] 0.69–2.68) or 90-day mortality (OR 1.22; 95% CI 0.62–2.39). Neither was fibrosis (i.e., periportal, septal, or biliary cirrhosis) associated with PHLF (OR 0.76; 95% CI 0.41–1.41) or 90-day mortality (OR 0.60; 95% CI 0.33–1.06). The independent risk factors for PHLF were preoperative cholangitis (OR 2.38; 95% CI 1. 36–4.17) and future liver remnant smaller than 40% (OR 2.40; 95% CI 1.31–4.38). The independent risk factors for 90-day mortality were age of 65 years or older (OR 2.40; 95% CI 1.36–4.23) and preoperative cholangitis (OR 2.25; 95% CI 1.30–3.87).

**Conclusion:**

In this study, no association could be demonstrated between hepatic steatosis or fibrosis and postoperative outcomes after resection of pCCA.

**Supplementary Information:**

The online version contains supplementary material available at 10.1245/s10434-023-14419-x.

The operative risk of curative-intent surgery for perihilar cholangiocarcinoma (pCCA) is among the highest for any elective procedure in oncologic surgery. A 43% severe morbidity rate and a 12% 90-day mortality rate after major liver resection for pCCA have been reported in Western studies.^1^ Posthepatectomy liver failure (PHLF) is the main cause for these fatalities.^2^ The liver is unique in its ability to regenerate in response to injury, such as liver resection.^3^ However, in the context of pre-existing liver disease, the ability of liver regeneration may be impaired.

Underlying hepatic parenchymal abnormalities, such as hepatic steatosis or fibrosis, pose new challenges for surgical patients. Non-alcoholic fatty liver disease (NAFLD) has become the most common chronic liver disease worldwide, and its global prevalence, currently estimated to be about 30%, is increasing.^4,5^ The underlying causes include the increasing prevalence of obesity and metabolic syndrome.^6^

The influence of hepatic steatosis on postoperative outcomes in liver surgery was first acknowledged in transplantation studies. The presence of severe steatosis in the liver graft (> 60%) was associated with a higher risk of graft failure and recipient death.^7,8^ Historically, liver biopsy was the gold standard for preoperative assessment of hepatic steatosis. Because of improved imaging techniques, guidelines currently recommend liver biopsy for potential living liver donors with unexplained abnormalities in liver function tests, body mass index (BMI) approaching 30 kg/m^2^, suspected 10% liver steatosis or more on imaging, or aspartate aminotransferase (AST) greater than alanine transaminase (ALT).^9,10^

Liver fibrosis, especially in an advanced stage (cirrhosis), is another pre-existent liver condition that may influence liver regeneration. Fibrosis is scar formation due to chronic or repeated injury replacing liver parenchyma.^11^ Cholestatic effects, for example caused by an obstructing tumor at the hepatic hilum, also may cause fibrosis.^12^ The severity of fibrosis was found to be related to PHLF in patients with hepatocellular carcinoma (HCC) undergoing right hemihepatectomy.^13^

For pCCA, most published studies have focused on the causal role of steatosis and fibrosis in the development of cholangiocarcinoma.^14^ To date, their role as a prognostic factor for postoperative outcomes for pCCA patients has not been clarified. Therefore, the first aim of this study was to determine the prevalence of hepatic steatosis and fibrosis among patients who underwent a resection for pCCA. The second aim was to determine the influence of hepatic steatosis and fibrosis on PHLF and 90-day postoperative mortality after major liver resection for pCCA.

## Methods

The study included all patients who underwent major liver resection for pCCA between 2000 and 2021 at three tertiary referral hospitals (University Hospital RWTH Aachen; Amsterdam UMC, location Academic Medical Center; and Erasmus MC, Rotterdam). The diagnosis was confirmed by histopathologic examination of the resected specimen. The exclusion criteria ruled out minor liver resection (i.e., < 4 segments resected), extrahepatic bile duct resection only, and liver transplantation. Clinical data were retrieved from prospectively maintained databases. The study was approved by the medical ethic committee at the Amsterdam UMC, location Academic Medical Center, and the need for informed consent was waived.

### Patient Workup and Management

Patient workup and management was comparable across all three centers. Assessment of the future liver remnant (FLR) was performed with computed tomography (CT)-volumetric assessment, hepatobiliary scintigraphy, or both using technetium-labeled mebrofenin (HBS). An FLR volume greater than 40% was generally considered sufficient. Portal vein embolization (PVE) was considered in other patients, but not performed when the optimal resection (left or right hemihepatectomy) was uncertain based on preoperative imaging.

### Definitions and Primary Outcomes

The primary outcomes were PHLF and 90-day postoperative mortality. Liver failure was scored and graded according to the International Study Group of Liver Surgery (ISGLS) criteria.^15^ Only grades B and C were considered clinically relevant. Overall survival (OS) was calculated from the date of surgery to the date of death from any cause or the last follow-up visit.

### Histologic Evaluation

Hepatic steatosis was defined as cytoplasmic accumulation of fat droplets affecting at least 5% of the hepatocytes.^16^ The severity of steatosis was based on the percentage of hepatocytes containing fat droplets as folows: none (< 5%), mild (5–30%), moderate (> 30–60%), or severe (> 60%). Liver fibrosis was described as no fibrosis, periportal fibrosis, septal fibrosis, or biliary cirrhosis.

All resected specimens were re-evaluated by one of two pathologists (M.D. or L.H.) for the presence of steatosis and fibrosis. Steatosis and fibrosis were assessed as distant as possible in relation to the tumor. Hematoxylin and eosin (H&E)-stained sections were used to evaluate steatosis. The presence and severity of fibrosis was evaluated by Sirius red (SR) or Elastica van Gieson (EvG) stains. If these connective tissue stains were not available, fibrosis was evaluated on H&E slides.

### Statistical Analysis

Statistical analysis was performed using SPSS Statistics (version 28.0.1.0; SPSS, IBM Corp., Armonk, NY). Categorical variables were presented as counts with percentages. Differences were tested with the chi-square or Fisher’s exact test. Continuous variables were described as medians with interquartile ranges (IQRs), and differences were tested using Mann Whitney *U* or Kruskal Wallis tests. Uni- and multivariable analyses were performed using binary logistic regression analysis.

All variables with a *p* value of 0.1 or lower were entered into the multivariable analyses using backward selection. A *p* value lower than 0.05 was considered statistically significant.

## Results

### Patient Characteristics

During the study period, 401 patients who underwent curative-intent major liver resection for pCCA were included. The median age was 65 years, and the majority of the patients were male (*n* = 253, 63.3%). One third of the patients were classified as American Society of Anesthesiology (ASA) III or IV (*n* = 132, 32.9%). About half of all resections were extended, with 62 patients (15.5%) undergoing an extended left hemihepatectomy and 147 patients (36.7%) undergoing an extended right hemihepatectomy. Preoperative PVE was performed for 104 patients (37%). For 61 patients (16%), PHLF (ISGLS grade B/C) occurred, and the overall 90-day mortality rate was 17% (*n* = 68). The 90-day mortality rate for the 39 patients with PHFL was 62%.

### Steatosis

Table 1 presents the baseline characteristics of the patients stratified according to steatosis grade (none vs mild vs moderate/severe). Steatosis was absent in 334 patients (83.3%), mild in 58 patients (14.5%), and moderate to severe in 9 patients (2.2%). The patients in the moderate (*n* = 7) and severe (*n* = 2) groups were combined due to the low number of affected patients. No ballooning or signs of steatohepatitis were observed.

**Table 1:** Baseline characteristics stratified according to steatosis grade

	All (*n* = 401)* n* (%)	None (*n* = 334)* n* (%)	Mild (*n* = 58)* n* (%)	Moderate/severe (*n* = 9)* n* (%)	*p* Value
Median age: years (IQR)	65 (57–71)	65 (56–71)	67 (60–72)	70 (63–71)	0.218
Male sex	253 (63)	215 (65)	36 (59)	2 (33)	0.220
Median BMI: ≥ 25 kg/m^2^ (IQR)	25 (22.5–26.7)	24.5 (22.3–26.4)	26 (24–29)	26 (23.8–28.7)	0.002
	196 (50)	152 (47)	38 (68)	6 (67)	0.009
ASA score					
I	56 (14)	47 (14)	8 (14)	1 (11)	0.917
II	210 (53)	174 (53)	30 (52)	6 (67)	
III	126 (32)	104 (31)	20 (35)	2 (22)	
IV	6 (2)	6 (2)	0 (0)	0 (0)	
Jaundice at presentation	302 (76)	255 (76)	40 (70)	7 (78)	0.598
Biliary drainage (ERCP, PTC, or both)	340 (85)	284 (86)	49 (85)	7 (78)	0.799
Preoperative cholangitis	156 (39)	132 (40)	21 (36)	3 (33)	0.814
PVE performed	104 (26)	94 (28)	8 (14)	2 (22)	0.067
FLR below 40 %	134 (37)	109 (36)	21 (40)	4 (57)	0.470
Surgery					
Left hemihepatectomy	108 (27)	86 (26)	20 (35)	2 (22)	0.349
Extended left hemihepatectomy	62 (16)	47 (14)	12 (21)	3 (33)	
Right hemihepatectomy	80 (20)	67 (20)	11 (21)	1 (11)	
Extended right hemihepatectomy	147 (37)	130 (39)	14 (24)	3 (33)	
Central/mesohepatectomy	4 (1)	4 (1)	0 (0)	0 (0)	
PV reconstruction	197 (50)	169 (51)	25 (44)	3 (33)	0.381
HA reconstruction	18 (5)	15 (5)	2 (3)	1 (11)	0.588
ISGLS liver failure (grade B/C)	63 (16)	50 (15)	11 (19)	2 (22)	0.647
30-Day mortality	52 (15)	43 (15)	8 (15)	1 (11)	0.957
90-Day mortality	68 (17)	55 (17)	10 (17)	3 (33)	0.412

*IQR* interquartile range, *BMI* body mass index, *ASA* American Society of Anesthesiologists, *ERCP* endoscopic retrograde cholangiopancreatography, *PTC* percutaneous transhepatic cholangiography, *PVE* portal vein embolization, *FLR* future liver remnant, *PV* portal vein, *HA* hepatic artery, *ISGLS* International Study Group of Liver Surgery

The baseline characteristics, treatment, and outcomes of each patient with moderate-to-severe steatosis are presented in Table S1. The BMI was 25 kg/m^2^ or higher for 47 % of the patients without steatosis, which was lower than 68% of the patients with mild steatosis and 67% of those with moderate-to-severe steatosis (*p* = 0.009). No differences in PHLF were observed between the patients without steatosis (15%), those with mild steatosis (19%), and those with moderate-to-severe steatosis (22%) (*p* = 0.647). No differences 90-day mortality rates could be demonstrated between the patients without steatosis (17%), the patients with mild steatosis (17%), and the patients with moderate-to-severe steatosis (33%) (*p* = 0.412).

### Fibrosis

Table 2 presents the baseline characteristics of the patients stratified according to fibrosis. Fibrosis was assessed based on H&E slides (*n* = 307) and connective tissue stains (*n* = 92). The study showed no fibrosis in 92 patients (23.1%), periportal fibrosis in 150 patients (37.6%), septal fibrosis in 123 patients (30.8%), and biliary cirrhosis in 34 patients (8.5%). For two patients, insufficient slides were available to assess fibrosis. The baseline characteristics, treatment, and outcomes for each patient with cirrhosis are presented in Table S2. The proportion of males was higher among the patients with septal fibrosis (74%) or biliary cirrhosis (71%) than among the patients without fibrosis or periportal fibrosis (both 57%; *p* = 0.014). Almost half of all the patients with cirrhosis (47.1%, 16/34) underwent a left hemihepatectomy, compared with only 25.2% (92/365) of the non-cirrhotic patients (*p* = 0.006).

**Table 2:** Baseline characteristics stratified according to fibrosis

Fibrosis	All (*n* = 399)* n* (%)	No fibrosis (*n* = 92)* n* (%)	Periportal fibrosis (*n* = 150)* n* (%)	Septal fibrosis (*n* = 123)* n* (%)	Biliary cirrhosis (*n* = 34)* n* (%)	*p* Value
Median age: years (IQR)	65 (57–71)	65 (56–71)	66 (58–72)	65 (57–71)	63 (58–72)	0.943
Male sex	252 (63)	52 (57)	86 (57)	90 (74)	24 (71)	0.014
Median BMI: kg/m^2^ (IQR)	25 (22.5–26.7)	24.7 (22.4–26.6)	24.5 (22.1–27.3)	25.3 (23–26.5)	25 (22.9–27)	0.216
ASA						
I	56 (14)	17 (19)	18 (12)	17 (14)	4 (12)	0.025
II	209 (53)	38 (41)	81 (54)	64 (53)	26 (79)	
III	125 (32)	34 (37)	48 (32)	40 (33)	3 (9)	
IV	6 (2)	3 (3)	3 (2)	0 (–)	0 (–)	
Jaundice at presentation	300 (75)	70 (77)	111 (74)	94 (76)	25 (74)	0.939
Biliary drainage (ERCP, PTC, or both)	338 (85)	76 (83)	128 85)	106 (87)	28 (85)	0.858
Preoperative cholangitis	155 (39)	32 (35)	56 (38)	52 (43)	15 (46)	0.560
PVE performed	104 (26)	25 (27)	48 (32)	29 (24)	2 (6)	0.018
FLR below 40 %	132 (37)	36 (45)	47 (34)	43 (39)	6 (20)	0.088
Surgery						
Left hemihepatectomy	108 (27)	22 (24)	41 (27)	29 (24)	16 (47)	0.234
Extended left hemihepatectomy	62 (16)	17 (19)	22 (15)	21 (17)	2 (6)	
Right hemihepatectomy	79 (20)	17 (19)	28 (19)	25 (20)	9 (27)	
Extended right hemihepatectomy	146 (37)	36 (39)	56 (37)	47 (38)	7 (21)	
Central/mesohepatectomy	4 (1)	0 (–)	2 (2)	1 (1)	0 (–)	
PV reconstruction	196 (50)	56 (61)	77 (52)	52 (43)	11 (32)	0.011
HA reconstruction	18 (5)	7 (8)	3 (2)	8 (7)	0 (–)	0.073
Complete resection (R0)	291 (76)	74 (84)	111 (76)	86 (74)	20 (63)	0.080
ISGLS liver failure (grade B/C)	62 (16)	17 (19)	18 (12)	23 (19)	4 (12)	0.353
30-Day mortality	52 (15)	15 (20)	13 (10)	18 (16)	6 (18)	0.223
90-Day mortality	67 (17)	21 (23)	17 (11)	21 (17)	8 (24)	0.082

*IQR* interquartile range, *BMI* body mass index, *ASA* American Society of Anesthesiologists, *ERCP* endoscopic retrograde cholangiopancreatography, *PTC* percutaneous transhepatic cholangiography, *PVE* portal vein embolization, *FLR* future liver remnant, *PV* portal vein, *HA* hepatic artery, *ISGLS* International Study Group of Liver Surgery

### Risk Factors for 90-Day Mortality and Liver Failure

Steatosis (≥ 5%) was not associated with PHLF (odds ratio [OR] 1.36; 95% confidence interval [CI] 0.69–2.68) or 90-day mortality (OR 1.22; 95% CI 0.62–2.39). Neither was fibrosis (i.e., periportal, septal, or biliary cirrhosis) associated with PHLF (OR 0.76; 95% CI 0.41–1.41) or 90-day mortality (OR 0.60; 95% CI 0.33–1.06). The independent risk factors for PHLF were preoperative cholangitis (OR 2.38; 95% CI 1.36–4.17) and FLR less than 40% (OR 2.40; 95% CI 1.31–4.38) (Table 3). The independent risk factors for 90-day mortality were age of 65 years or older (OR 2.40; 95% CI 1.36–4.23) and preoperative cholangitis (OR 2.25; 95% CI 1.30–3.87) (Table 4). Hepatic steatosis and fibrosis were not independent risk factors for PHLF or 90-day mortality.

**Table 3:** Multivariable analysis of predictors for PHLF (ISGLS grade B/C)

	Univariable analysis			Multivariable analysis		
	OR	95% CI	*p* value	OR	95% CI	*p* value
Age ≥65 years	1.40	0.81–2.41	0.228			
Male sex	1.54	0.85–2.77	0.152			
BMI ≥ 25 kg/m^2^	1.08	0.62–1.89	0.788			
ASA score						
I/II	Ref	–	–			
III/IV	1.29	0.74–2.26	0.366			
Jaundice at presentation						
Biliary drainage	2.86	1.26–6.52	0.012			
Any	4.00	1.21–13.21	0.023			
Preoperative cholangitis	2.39	1.38–4.13	0.002	2.38	1.36–4.17	0.002
FLR below 40%	2.71	1.52–4.82	<0.001	2.40	1.31–4.38	0.005
PV reconstruction	0.79	0.46–1.36	0.395			
HA reconstruction	1.55	0.49–4.89	0.450			
Hepatic steatosis						
Grade 0 (< 5 %)	Ref	–	–	Ref	–	–
Grades 1–3 (≥ 5 %)	1.36	0.69–2.68	0.370	1.36	0.67–2.73	0.395
Hepatic fibrosis						
None	Ref	–	–	Ref	–	–
Fibrosis^a^	0.76	0.41–1.41	0.383	0.76	0.40–1.45	0.408

*PHLF* posthepatectomy liver failure, *ISGLS* International Study Group of Liver Surgery, *OR* odds ratio, *CI* confidence interval, *BMI* body mass index, *ASA* American Society of Anesthesiologists, *FLR* future liver remnant, *PV* portal vein, *HA* hepatic artery

^a^Periportal fibrosis, septal fibrosis, or biliary cirrhosis

**Table 4:** Multivariable analysis of predictors for 90-day mortality

	Univariable analysis			Multivariable analysis		
	OR	95% CI	*p* value	OR	95% CI	*p* value
Age ≥65 years	2.39	1.37–4.18	0.002	2.40	1.36–4.23	0.002
Male sex	1.37	0.78–2.40	0.272			
BMI ≥ 25 kg/m^2^	1.42	0.82–2.44	0.211			
ASA score						
I/II	Ref	–	–			
III/IV	2.11	1.24–3.60	0.006			
Jaundice at presentation	2.35	1.12–4.94	0.024			
Biliary drainage						
Any	2.41	0.93–6.27	0.072			
Preoperative cholangitis	2.12	1.24–3.62	0.006	2.25	1.30–3.87	0.004
FLR below 40 %	1.87	1.08–3.25	0.026			
PV reconstruction	1.57	0.93–2.67	0.093			
HA reconstruction	1.42	0.45–4.44	0.552			
Hepatic steatosis						
Grade 0 (< 5 %)	Ref	–	–	Ref	–	–
Grades 1–3 (≥ 5 %)	1.22	0.62–2.39	0.559	1.15	0.57–2.30	0.701
Hepatic fibrosis						
None	Ref	–	–	Ref	–	–
Fibrosis^a^	0.60	0.33–1.06	0.080	0.56	0.31–1.01	0.055

*OR* odds ratio, *CI* confidence interval, *BMI* body mass index, *ASA* American Society of Anesthesiologists, *FLR* future liver remnant, *PV* portal vein, *HA* hepatic artery

^a^Periportal fibrosis, septal fibrosis, or biliary cirrhosis

## Discussion

In this study of 401 patients who underwent a resection for pCCA, the incidence of any steatosis (> 5% hepatocytes involved) was 16.7%, and the incidence of moderate-to-severe steatosis (> 30%) was only 2.2%. Hepatic cirrhosis was found in 8.5% of the patients. No association could be demonstrated between the presence of steatosis or fibrosis and PHLF or 90-day mortality. The independent risk factors for PHLF were preoperative cholangitis and FLR less than 40%, and the independent risk factors for 90-day mortality were age of 65 years or older and preoperative cholangitis.

Approximately 25–30% of the Western adult population is affected by hepatic steatosis, with a rising prevalence.^17–19^ The prevalence of NAFLD varied from 0.8 to 44.1% in a systematic review and meta-analysis that aimed to determine a potential association between NAFLD and cholangiocarcinoma.^14^ The differences in diagnostic methods for NAFLD accounted for the significant variation observed among studies. The highest prevalence was recorded in studies that used histopathologic examination for diagnosis (15.6–69.3%), followed by imaging (20.0–30.9%) and elevated liver enzyme levels (7.9–14.9%).^20^ A few studies reported hepatic steatosis based on histopathologic assessment of patients undergoing liver resection. Moderate-to-severe hepatic steatosis was found in 18% of 386 patients undergoing hepatic resection for colorectal liver metastasis (CRLM), which was much higher than the 2.2% in the current study.^21^ In a similar study, moderate-to-severe steatosis was present in 14.9% of the 934 included patients.^22^ A possible explanation might be that patients with CRLM more often received neo-adjuvant chemotherapy, which can induce hepatic steatosis.^23^

The influence of steatosis on postoperative outcomes after liver surgery has been a topic of debate. In the current study, hepatic steatosis was not associated with postoperative 90-day mortality or PHLF. Opposite results were found in a systematic review and meta-analysis of steatosis and post-hepatectomy outcomes. In that review, the patients with hepatic steatosis (> 30%) had a twofold increased risk of postoperative complications and almost a threefold increased risk of mortality versus the risk for patients without hepatic steatosis.^24^ Only four studies with a total of 1000 patients published more than 15 years ago were included in the analysis. Behrns et al.^25^ concluded that moderate-to-severe steatosis may be associated with postoperative mortality based on two postoperative deaths among seven patients. Kooby et al.^26^ matched patients with normal liver to those with steatotic liver based on only three variables (age, comorbidity, and extent of liver resection). The presence of steatosis was associated with postoperative complications (any), but not with mortality. Gomez et al.^21^ found that severity of steatosis was an independent predictor of postoperative morbidity in 386 patients undergoing resection for CRLM. All four studies included patients undergoing major hepatectomy for “hepatic neoplasms,” which led to a heterogeneous cohort.^21,25–27^

A more recent study included in the meta-analysis analyzed about 1000 liver resections for CRLM and found no differences in 90-day mortality between steatotic (> 5%) and non-steatotic (≤ 5%) patients (steatosis [3.3%] vs no steatosis [2.7%]; *p* = 0.595), or between different steatosis grades (mild [3.5%], moderate [3%], severe [2.6%]; *p* = 0.931).^22^ In 152 of 996 HCC patients, NAFLD was associated with an increased rate of major morbidity (16.2% vs 8.1%; *p* < 0.001) and a higher rate of PHLF grade B/C (20.1% vs 7.2% grade B/C; *p* < 0.001).^28^ A propensity score-matching analysis of 863 patients with fatty livers and 863 patients with normal livers showed that the patients with fatty livers had increased major morbidity (32% vs 24%; *p* = 0.001), but similar PHLF (5.9% vs 4.4%; *p* = 0.157) and mortality (2.7% vs 1.9%; *p* = 0.257).^29^ However, the definition of “fatty liver” was not further specified, and histology assessment was not available.

Liver cirrhosis is a significant contributor to morbidity and mortality on a global scale.^30^ Among 1001 patients undergoing liver resection for various indications, cirrhosis was present in 12%.^31^ The presence of cirrhosis in hepatobiliary disease is dependent on the malignancy. In a recent meta-analysis, cirrhosis was present in at least half of all the patients who underwent hepatic resection for HCC.^32^ In contrast to intrahepatic cholangiocarcinoma (iCCA), extrahepatic cholangiocarcinoma is less frequently reported for patients with a background of liver cirrhosis.^33^ In two large population studies, liver cirrhosis was found in 8–10% of iCCA patients compared with 4% of 539 patients who underwent a resection for pCCA.^34–36^ The incidence of cirrhosis in the current study (8.5%) was similar.

Hepatic fibrosis as a risk factor for PHLF and postoperative mortality has not been studied specifically for pCCA. Previous studies that compared cirrhosis with no cirrhosis primarily included patients with HCC. Functional reserve and hepatic perfusion are impaired due to pathologic alternations in the liver, which leads to decreased hepatocyte function and regeneration capacity.^37–39^ A systematic review found that patients with cirrhosis showed less hypertrophy response to PVE than patients with a normal liver.^40^ An FLR of at least 40% is recommended for patients with cirrhosis.^41,42^

The current study found that factors other than steatosis or fibrosis were risk factors for PHLF and 90-day mortality. Preoperative cholangitis was an independent predictor of both outcomes, whereas FLR below 40% was predictive of PHLF, and age of 65 years or older was predictive of 90-day mortality. The FLR volume is often considered the most important predictor of PHLF.^2,43,44^ In a previous study, the following five independent risk factors for 90-day mortality were included in a risk score: age, preoperative cholangitis, FLR volume less than 30%, portal vein reconstruction, and incomplete FLR drainage in patients with an FLR volume less than 50%.^45^ A risk score for PHLF found that preoperative jaundice, preoperative cholangitis, FLR volume, and preoperative bilirubin were independent risk factors.^2^

Preoperative cholangitis remains a major concern in the management of patients with pCCA. Preoperative biliary drainage (PBD) is required for patients with obstructive cholangitis. However, PBD in the absence of cholangitis is a subject of debate because it may potentially increase the risk of PHLF and mortality due to associated cholangitis. No consensus exists on how to drain the bile ducts, whether by percutaneous transhepatic biliary drainage (PTBD), endoscopic biliary drainage (EBD), or both.^46^ For patients with insufficient FLR (i.e., below 40%), PBD is required before PVE because liver regeneration is impaired due to biliary obstruction. For patients with an FLR volume greater than 50% (e.g., a left hemihepatectomy), the potential benefits of PBD do not justify the risk of cholangitis and related mortality after drainage.^45,47^

The current guidelines for living donor liver transplant (LDLT) do not consider liver biopsy as a standard procedure for evaluating hepatic steatosis.^9,10^ Surgical risk for recipients has been established in steatotic donors. The presence of moderate-to-severe steatosis (> 30%) was associated with initial poor graft function (*n* = 12 [17%] vs *n* = 17 [35%]; *p* < 0.03) compared with the presence of mild steatosis (< 30%).^8^ Unacceptable risk of graft loss within 1 year occurred more frequently for patients with moderate-to-severe steatosis than for non-steatotic patients (*n* = 73 [22.5%] vs *n* = 18 [37.5%]; *p* < 0.03).^8^ Of 101 patients undergoing hepatic biopsies in the workup of living donors for right lobe liver transplantation, 33% had some degree of steatosis (> 5%), and five potential donors had more than 30% hepatic steatosis.^48^ In that study, 12 potential donors were denied based on liver biopsy, 7 potential donors had significant steatosis or graft adjusted to recipient body weight less than 0.8%, 3 donors were rejected because of unexpected findings, and 2 donors were rejected because of medical circumstances. To date, the exact cutoff value for acceptable steatosis in LDLT is not well-defined, but acceptable limits range from 10 to 30%, depending on other factors such as liver volume and right or left lobe donation.^10^ No studies were found that performed preoperative liver biopsies to evaluate steatosis or fibrosis in pCCA patients. Percutaneous liver biopsy is not without risk. In one study, major complications (i.e., bleeding, injury to adjacent organs, sepsis) occurred in 2.4% of the patients, with a mortality rate of 0.01%.^49^

This study had several limitations. First, histologic assessment was performed on the resected liver specimen rather than a biopsy of the remnant liver. The resected liver may have been more affected by biliary obstruction and atrophy, which may have biased assessment of fibrosis and steatosis. Also, the PVE may have affected the degree of fibrosis. However, obstructive-type ductular reactions can rapidly resolve after the obstruction is relieved.^50^ Second, in most of the patients, the degree of fibrosis was evaluated based on the H&E slides instead of a connective tissue stain, which is recommended for assessing fibrosis. Third, the incidence of moderate-to-severe steatosis was low, which resulted in wide confidence intervals for PHLF and 90-day mortality. This limited the ability to analyze the association of severe-to-moderate steatosis with worse outcomes. A much larger dataset is required to rule out a clinically relevant association of steatosis and cirrhosis with postoperative outcomes. Finally, the presence of steatosis in the study cohort was lower than in the general population. Selection bias may have occurred because the study included only patients who underwent a resection, and patients with severe steatosis on imaging may not have been considered for resection.

In conclusion, no association could be demonstrated between hepatic steatosis or fibrosis and postoperative outcomes after resection of pCCA in this study. Advanced age, FLR lower than 40%, and preoperative cholangitis were independent risk factors for PHLF and 90-day mortality.

### Supplementary Information

Below is the link to the electronic supplementary material.

Supplementary file1 (DOCX 35 kb)
