# Supplementary material for: The Influence of Hepatic Steatosis and Fibrosis on Postoperative Outcomes After Major Liver Resection of Perihilar Cholangiocarcinoma
Source: Ann Surg Oncol. 2023 Oct 29;31(1):133–41. doi: 10.1245/s10434-023-14419-x (PMC10695871; doi:10.1245/s10434-023-14419-x)
Supplement: Supplementary file 1 — Supplementary file1 (DOCX 35 kb) [file 10434_2023_14419_MOESM1_ESM.docx]

**Supplementary Table 1** Characteristics of 9 patients with moderate/severe steatosis

| **Steatosis** | **Gender** | **Age** | **BMI** | **ASA** | **Bismuth** | **Biliary drainage** | **Preoperative cholangitis** | **PVE** | **Preoperative FLR%** | **Resection type** | **Liver failure ISGLS** | **90-day mortality** |
| --- | --- | --- | --- | --- | --- | --- | --- | --- | --- | --- | --- | --- |
| Moderate | Male | 57 | 26 | 2 | 3A | ERCP | No | Yes | 28% | Right Extended Hemi | none | No |
| Moderate | Female | 62 | 23 | 2 | 3B | PTC | No | No | 63% | Left Extended Hemi | none | Yes (sepsis) |
| Moderate | Female | 64 | 24 | 2 | 3B | No | No | No | 36% | Left Extended Hemi | None | No |
| Moderate | Female | 68 | 44 | 3 | 3A | ERCP | No | No | 47% | Left Extended Hemi | none | No |
| Moderate | Female | 70 | 29 | 3 | 4 | Both | No | Yes | 36% | Right Extended Hemi | none | No |
| Moderate | Female | 71 | 28 | 2 | 3A | ERCP | Yes | No | 24% | Right Extended Hemi | B | Yes (sepsis) |
| Moderate | Male | 75 | 26 | 1 | 3A | Both | Yes | No |  | Right hemi | B | Yes (sepsis) |
| Severe | Female | 70 | 25 | 2 | 2 | None | No | No | 72% | Left Hemi | none | No |
| Severe | Male | 71 | 24 | 2 | 3B | Both | Yes | No | *NA* | Left Hemi | A | No |

Abbreviations*: BMI,* body mass index; *ASA,* American Society of Anesthesiologists; *NA,* not available; PVE*,* portal vein embolization; FLR, future liver remnant; *ISGLS,* International Study Group of Liver Surgery.

**Supplementary Table 2** Characteristics of 34 patients with cirrhosis

| **Gender** | **Age** | **BMI** | **ASA** | **Bismuth** | **Biliary drainage** | **Preoperative cholangitis** | **Resection type** | **PVE** | **Preoperative FLR%** | **Liver failure ISGLS** | **90-day mortality** |
| --- | --- | --- | --- | --- | --- | --- | --- | --- | --- | --- | --- |
| Female | 48 | 18 | 2 | 3B | Both | Yes | Left Hemi | No | 81% | None | No |
| Female | 49 | 24 | 2 | 4 | Both | Yes | Left Hemi | No | *NA* | None | No |
| Male | 55 | 24 | 2 | 3A | ERCP | No | Right Extended Hemi | No | 47% | None | No |
| Male | 55 |  | 2 | 3B | ERCP | No | Left Hemi | No | 76% | A | No |
| Male | 55 | 27 | 2 | 3A | ERCP | No | Right Extended Hemi | No | 40% | A | No |
| Male | 56 | 29 | 2 | 4 | ERCP | Yes | Left Extended Hemi | No | *NA* | None | No |
| Female | 57 | 22 | 1 | 3A | None | No | Right Hemi | No | 48% | None | Yes |
| Male | 58 | 24 | 1 | 4 | ERCP | No | Left Extended Hemi | No | 84% | None | No |
| Male | 58 | 19 | 2 | 3B | None | No | Left Hemi | No | 74% | None | No |
| Male | 58 | 29 | 2 | *NA* | None | No | Right Hemi | No | 43% | None | No |
| Female | 59 | 26 | 2 | 3A | ERCP | No | Right Extended Hemi | No | 36% | None | No |
| Male | 60 | 30 | 2 | 3A | Both | Yes | Right Hemi | Yes | 38% | B | Yes |
| Male | 61 | 25 | 1 | 4 | ERCP | No | Left Hemi | No | 82% | None | No |
| Male | 61 | 27 | 2 | 3B | ERCP | No | Left Hemi | No | 78% | None | No |
| Male | 61 | 25 | 2 | 3A | ERCP | Yes | Right Hemi | No | 43% | None | No |
| Female | 62 |  | 2 | 3A | ERCP | No | Right Extended Hemi | No | 30% | None | No |
| Female | 62 | 28 | 2 | 2 | ERCP | No | Right Hemi | No | 44% | None | No |
| Male | 64 |  | 2 | 3B | Both | Yes | Left Hemi | No | 66% | None | No |
| Male | 64 | 23 | 2 | 3A | Both | Yes | Right Extended Hemi | Yes | 17% | A | No |
| Male | 66 | 23 | 2 | 3B | PTC | No | Left Hemi | No | 75% | None | No |
| Male | 67 | 26 | 3 | 3B | ERCP | No | Left Hemi | No | 88% | B | Yes |
| Female | 67 | 27 | 3 | 3B | ERCP | Yes | Left Hemi | No | 78% | None | No |
| Female | 69 | 25 | 2 | 3B | Both | Yes | Left Hemi | No | 74% | None | No |
| Male | 69 | 20 | 2 | 4 | Both | Yes | Left Hemi | No | 78% | None | No |
| Male | 71 | 27 | 2 | 3A | Both | Yes | Right Extended Hemi | No | 45% | None | No |
| Male | 72 | 20 | 2 | 3A | None | No | Right Hemi | No | 45% | None | No |
| Male | 72 | 25 | 3 | 3B | Both | Yes | Left Hemi | No | 84% | B | Yes |
| Male | 73 | 22 | 2 | 4 | ERCP | Yes | Left Hemi | No | 82% | None | No |
| Female | 74 | 24 | 2 | 3A | ERCP | Yes | Right Hemi | No | *NA* | C | Yes |
| Female | 74 | 22.4 | *NA* | *NA* | *NA* | *NA* | Right Extended Hemi | *NA* | 35% | *NA* | Yes |
| Male | 76 | 25 | 2 | 3B | ERCP | No | Left Hemi | No | 89% | None | Yes |
| Male | 78 | 26 | 1 | 3A | None | No | Right Hemi | No | *NA* | None | No |
| Male | 78 | 29 | 2 | 3A | ERCP | Yes | Right Hemi | No | 37% | None | Yes |
| Male | 79 | 24 | 2 | 3A | Both | No | Left Hemi | No | 84% | None | No |

Abbreviations*: BMI,* body mass index; *ASA,* American Society of Anesthesiologists; *NA,* not available; PVE*,* portal vein embolization; FLR, future liver remnant; *ISGLS,* International Study Group of Liver Surgery.
